# Comparative Evaluation of ChatGPT-5.2, Claude Sonnet 4.5, and DeepSeek-V3.2 for Rosacea-Related Information: Accuracy, Reliability, Readability, and Reference Hallucinations

**DOI:** 10.3390/diagnostics16162620

**Published:** 2026-08-18

**Authors:** Mahmut Talha Uçar, Ecem Bostan, Tülay Ortabağ, Elif Dönmez

**Affiliations:** 1 Saray District Health Directorate, Tekirdağ Provincial Health Directorate, Republic of Türkiye Ministry of Health, Tekirdağ 59600, Türkiye; 2 Dermatology and Venereology Department, Faculty of Medicine, Ankara Medipol University, Ankara 06050, Türkiye; bostanecem@gmail.com; 3 Dermatology and Venereology Department, Private Bilgi Hospital, Ankara 06050, Türkiye; 4 Department of Nursing, Faculty of Health Sciences, Istanbul Topkapi University, Istanbul 34087, Türkiye; tulayortabag@topkapi.edu.tr; 5 Department of Oncology Nursing, Hamidiye Faculty of Nursing, University of Health Sciences, Istanbul 34668, Türkiye; ed.elifdonmez@gmail.com

**Keywords:** clinical decision support, generative artificial intelligence, diagnostic information, large language models, rosacea

## Abstract

**Background/Objectives:** Rosacea is a chronic inflammatory skin disease that requires long-term management and continuous patient education regarding triggers, skincare practices, and treatment adherence. In recent years, patients have increasingly turned to online platforms and artificial intelligence (AI)-based chatbots for health-related information. Although ChatGPT has been evaluated in the context of rosacea, evidence regarding the performance of other AI chatbots remains limited. This study aimed to evaluate the accuracy, reliability, quality, readability, and diagnostic relevance of AI-generated responses to common rosacea-related patient questions and to assess their potential role as sources of health-related information. **Methods:** Between 21 December 2025 and 22 February 2026, rosacea-related questions were collected from the publicly accessible Quora platform using a systematic screening process. Twenty clinically relevant and representative questions covering diagnosis, triggers, treatment options, skincare practices, and disease manifestations were selected. Each question was independently submitted to three AI chatbots (Claude Sonnet 4.5, ChatGPT-5.2, and DeepSeek-V3.2). Responses were evaluated by domain experts using the modified DISCERN (mDISCERN) for reliability, the Global Quality Scale (GQS) for overall quality, the Flesch Reading Ease Score (FRES) for readability, and a 5-point Likert scale for accuracy. Reference hallucinations were assessed through manual verification of cited sources. Statistical comparisons were performed using the Friedman test with Bonferroni-adjusted post hoc analyses, and effect sizes were calculated using Kendall’s coefficient of concordance (Kendall’s W). **Results:** Significant differences were observed among the AI chatbots across all evaluation domains (*p* < 0.05), with moderate to large effect sizes (Kendall’s W = 0.272–0.683). ChatGPT-5.2 and DeepSeek-V3.2 demonstrated significantly higher reliability and accuracy scores than Claude Sonnet 4.5. DeepSeek-V3.2 achieved the highest overall quality scores, whereas ChatGPT-5.2 produced the most readable responses. Reference analysis revealed variable hallucination rates among the evaluated models. **Conclusions:** Generative AI chatbots demonstrate considerable potential as sources of health-related information for rosacea-related queries. However, variability in performance and reference hallucination rates highlights the need for careful validation before their widespread use as complementary sources of patient health information.

## 1. Introduction

Rosacea is a common, chronic inflammatory skin condition characterized by centrofacial erythema, papules, pustules, and telangiectasia [1,2]. Although the precise pathophysiology of rosacea has not yet been fully elucidated, multiple contributing mechanisms have been proposed, including genetic susceptibility, alterations in innate and adaptive immune responses, neurovascular abnormalities, and changes in the cutaneous microbiome [1,2].

Because rosacea predominantly involves the facial area, it may negatively affect patients’ emotional well-being, leading to feelings of embarrassment, anxiety, reduced self-confidence, and depressive symptoms. Moreover, clinical manifestations such as papules, pustules, and erythema can contribute to physical discomfort [2]. Patients with rosacea often experience difficulties coping with the condition and are at increased risk of psychological problems such as depression, anxiety, stress, and suicidality, which substantially impair their quality of life [1]. The experiences of patients with rosacea can be categorized into several stages, including the prediagnostic phase, diagnosis, treatment, treatment adherence, and long-term disease management. During the prediagnostic phase, symptoms are frequently misinterpreted as acne or sunburn, resulting in diagnostic delays that may extend for months or even years. Following diagnosis, patients often face significant challenges related to selecting appropriate therapies, the chronic nature of treatment, and potential adverse effects [3]. In this context, it is essential to adequately address patients’ concerns and provide clear, comprehensive information.

Generative artificial intelligence (AI) systems have become increasingly integrated into everyday life as sources of information across diverse domains. By leveraging large-scale datasets, generative AI is capable of producing original outputs, including text, images, music, and video. Consequently, AI-powered chatbots are increasingly being used by patients as preliminary sources of medical information before seeking professional healthcare [4,5].

Patient education is a crucial component of rosacea management, particularly regarding trigger avoidance, skincare practices, and treatment adherence. Patients’ knowledge and understanding of rosacea may directly influence treatment-related decisions, including adherence to prescribed therapies, avoidance of disease triggers, expectations regarding treatment outcomes, and timely healthcare seeking. Inadequate or inaccurate information may contribute to inappropriate self-management and suboptimal treatment decisions [3,6]. Because many patients increasingly rely on online resources before consulting healthcare professionals, the quality and accuracy of health information can substantially influence their perceptions of disease severity, treatment expectations, and healthcare decision-making. Inaccurate or misleading information may lead to inappropriate self-management, delayed medical consultation, unnecessary anxiety, or poor adherence to evidence-based treatment recommendations [3,6,7]. Patients may not always receive sufficient information during routine clinical encounters. Consequently, AI-based chatbots have emerged as complementary patient education tools capable of providing personalized, on-demand health information and supporting patient engagement and self-management. Nevertheless, concerns regarding response accuracy, consistency, patient safety, and ethical considerations underscore the need for careful evaluation before their integration into routine patient care [7,8,9]. Online health information seeking has become increasingly common worldwide. Recent evidence indicates that 74.4% of adults in the United States search for health information online, while the prevalence exceeds 70% in several European countries and reaches 79–86% in some Asian populations, emphasizing the importance of ensuring the quality and reliability of online medical information [10]. As access to online health information increases, health literacy has become an increasingly important determinant of patients’ ability to obtain, understand, evaluate, and apply health information. Individuals with limited health literacy may have greater difficulty distinguishing accurate information from misinformation, making the readability and quality of online resources particularly important. Accordingly, the NIH, the U.S. Department of Health and Human Services, and the American Medical Association recommend that patient educational materials be written at approximately a sixth-grade reading level to maximize public comprehension. Consequently, readability should be regarded as an essential quality indicator when evaluating AI-generated health information [11,12]. Because rosacea affects a broad and diverse patient population, digital health information should be understandable and accessible to individuals with varying levels of health literacy, educational backgrounds, and reading abilities. Presenting medical information in clear and comprehensible language is essential to facilitate informed decision-making, improve treatment adherence, and reduce the risk of misunderstanding or misuse of health information [9,11,12]. As a result, many individuals increasingly turn to online platforms, social media, and AI-based tools to obtain information about rosacea. Nevertheless, the accuracy and validity of information provided by these sources remain a matter of ongoing debate.

Yan et al. evaluated the reliability and clinical applicability of ChatGPT in answering common rosacea-related questions and reported high reliability and applicability rates [13]. However, while studies evaluating ChatGPT in the context of rosacea are available, no research has systematically compared multiple contemporary AI chatbots in this field [13,14]. Recent bibliometric studies have demonstrated the rapid expansion and evolving landscape of rosacea research, highlighting major research hotspots, influential publications, and emerging trends over time. While traditional citation-based analyses remain essential for evaluating scientific impact, alternative metrics (Altmetrics) provide complementary insights into the online visibility and public engagement of scientific publications through social media and digital platforms. Together, these approaches improve our understanding of how scientific evidence is disseminated, reinforcing the importance of evaluating the quality and reliability of AI-generated health information available to patients [15,16].

Many patient queries regarding rosacea involve symptom recognition, differential diagnosis, treatment selection, trigger identification, and disease prognosis. As generative AI systems are increasingly used as sources of preliminary healthcare information prior to professional consultation, it is important to evaluate the quality and reliability of the information they provide. Therefore, this study aimed to evaluate the accuracy, reliability, quality, readability, and diagnostic relevance of AI-generated responses to common rosacea-related patient questions and to assess their potential as complementary sources of healthcare information.

## 2. Materials and Methods

This cross-sectional comparative study evaluated the accuracy, reliability, quality, readability, and reference hallucinations of AI-generated responses to rosacea-related patient questions. For the present study, ethical approval was not required because publicly accessible online data was used without human participants or patient records.

Patient-generated questions were collected from Quora (Quora, Inc., Mountain View, CA, USA), a publicly accessible question-and-answer platform, by searching the keyword “rosacea” and applying the “Questions” filter. Results were screened using the platform’s default relevance-based ranking, with no time restriction applied. Quora was selected because it is one of the largest publicly accessible question-and-answer platforms, providing a large volume of patient-generated health questions. To ensure a standardized and reproducible sampling frame while minimizing selection bias associated with arbitrary manual sampling, the first 200 questions returned by Quora’s default relevance-based ranking were systematically screened using predefined inclusion and exclusion criteria. This predefined threshold was selected to capture a broad spectrum of patient queries while maintaining feasibility for detailed expert review. Questions were excluded if they met any of the following predefined criteria: (i) not related to rosacea or dermatological conditions (e.g., botany-related “Rosaceae” content or non-medical topics), (ii) non-clinical or non-informational content (e.g., celebrity speculation or purely social commentary), (iii) promotional or advertising-oriented questions, (iv) duplicated or near-duplicated questions, or (v) absence of a clearly articulated health-related inquiry.

Following the initial screening, eligible questions were evaluated for clinical relevance and thematic diversity. A preliminary shortlist of 40 questions was generated by prioritizing questions that reflected patient information needs commonly encountered in routine clinical practice and by ensuring coverage across major thematic domains, including diagnosis, symptom recognition, triggers, treatment options, skincare practices, ocular involvement, and disease course.

**Figure 1:**
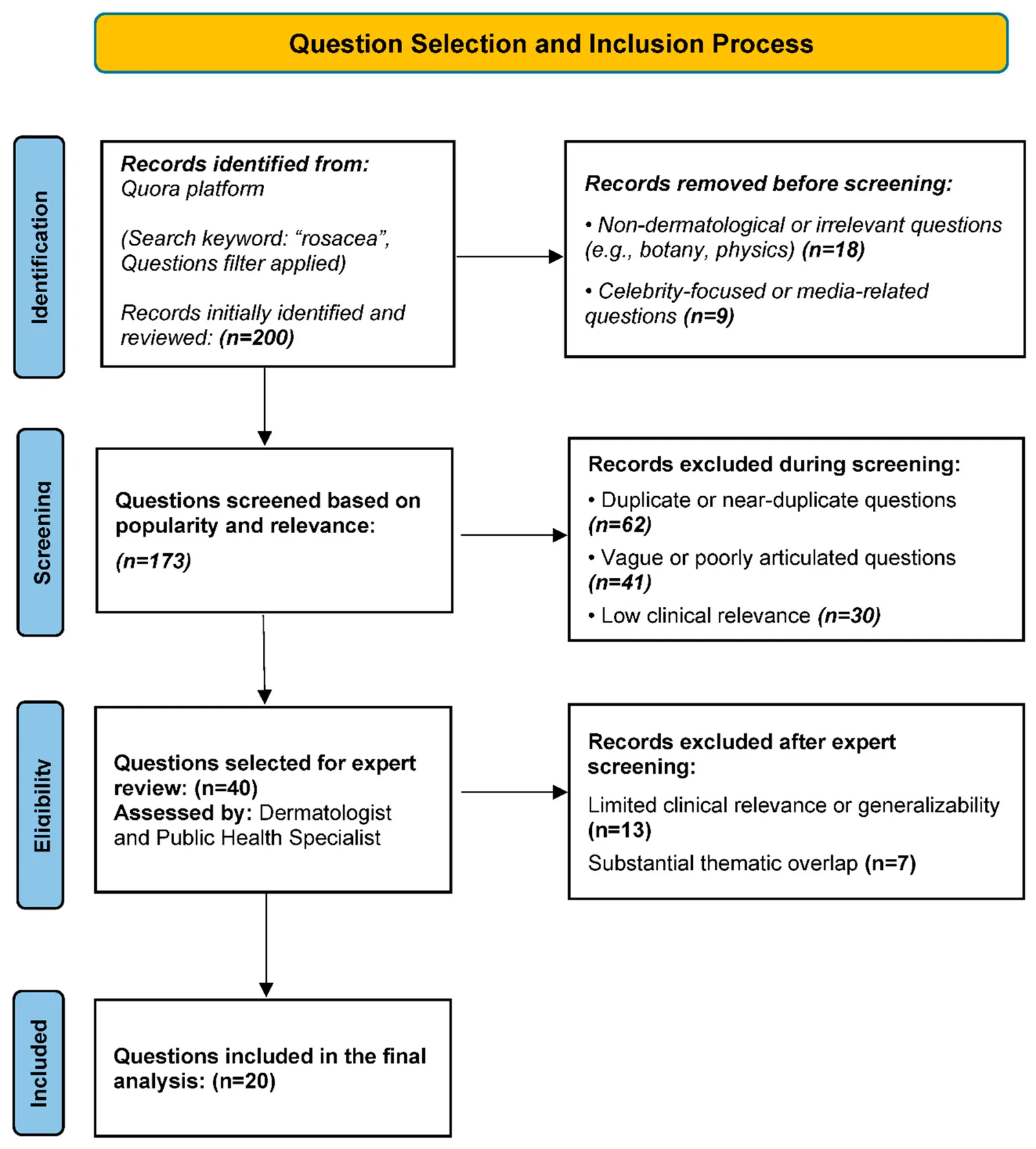
PRISMA-based flow diagram demonstrating the identification, screening, eligibility assessment, and inclusion of rosacea-related questions retrieved from Quora.

The shortlisted questions were independently reviewed by a board-certified dermatologist and a public health researcher. The final questions were selected to represent the major domains of patient information needs related to rosacea, including disease etiology, diagnosis, symptoms, triggers, treatment, skincare, prognosis, and disease management, while avoiding redundant questions addressing the same topic (Figure 1). The final 20 questions included in the analysis are presented in Table 1, whereas the complete list of the 40 shortlisted candidate questions is provided in Supplementary Table S1 to enhance the transparency and reproducibility of the selection process. Because no established sample size calculation method exists for expert-based comparative evaluations of large language model responses, the final sample of 20 representative questions was selected pragmatically to provide adequate coverage of the major clinical domains while maintaining feasibility for detailed expert assessment.

## 3. Statistical Analysis

Statistical analyses were performed using IBM SPSS Statistics version 27.0 (IBM Corp., Armonk, NY, USA). Descriptive statistics are presented as mean ± standard deviation (SD). Because the same set of questions was evaluated across all AI models, comparisons were performed using nonparametric tests for related samples. Differences in reliability (mDISCERN), quality (GQS), readability (FRES), and accuracy scores among the three models were assessed using the Friedman test, with mean ranks reported for each model. When significant differences were identified, pairwise comparisons were conducted using the Wilcoxon signed-rank test with Bonferroni correction (adjusted α = 0.0167). All tests were two-tailed, and *p*-values < 0.05 were considered statistically significant unless otherwise specified. In addition to statistical significance testing, effect sizes were calculated using Kendall’s coefficient of concordance (Kendall’s W) for Friedman test results. Values of approximately 0.1, 0.3, and 0.5 were interpreted as small, moderate, and large effect sizes, respectively.

### 3.1. AI Prompting and Response Collection

This study was conducted as a comparative descriptive evaluation of responses generated by artificial intelligence-based conversational models to rosacea-related health questions. The methodology was designed to reflect real-world user interaction patterns and to minimize bias related to prompt formulation.

All models were queried using identical, single-sentence clinical questions (e.g., “How do you reduce rosacea flare-ups?”). The prompts were intentionally kept simple and directive, without requesting references, citations, or guideline-based evidence. This approach was chosen because typical users primarily seek direct explanatory information at first contact and do not usually request sources during the initial query. Including reference requests at this stage was considered likely to influence response length, tone, and complexity.

Each question was submitted in a new, independent session for every model to prevent contextual carryover effects. To minimize potential personalization bias associated with browser history or user-specific algorithms, all prompts were submitted through the official websites of each model using the web browser’s private (incognito) mode. Because some platforms imposed limits on the number of queries that could be submitted within a given period, data collection was completed over multiple days as access became available. The primary responses obtained from this step constituted the main dataset for analysis and were evaluated for content clarity, medical accuracy, and informational quality.

After completion of the primary response, a separate follow-up question was asked to the same model requesting disclosure of external sources used to generate the response. Source attribution was therefore assessed independently from the initial informational output and was not included in the evaluation of first-level responses.

This two-step questioning strategy ensured that the primary answers reflected the type of information typically encountered by users in real-world settings while still allowing structured assessment of source transparency as a secondary outcome.

“For the information provided above, please provide the full bibliographic citations (including authors, publication year, title, and journal/source) for the specific external references used. If applicable, provide the direct URL link in plain text as well.”

To ensure structural consistency across all models and to facilitate qualitative content analysis, the models were instructed to provide their responses in plain text format only. In rare instances where a model attempted to generate tabular data, a standardized instruction was used to request the information in narrative form. This measure was taken to maintain a uniform layout for subsequent readability and accuracy assessments.

ChatGPT-5.2 (OpenAI, San Francisco, CA, USA), Claude Sonnet 4.5 (Anthropic PBC, San Francisco, CA, USA), and DeepSeek-V3.2 (DeepSeek AI, Hangzhou, China) were selected because they are representative of some of the most widely used contemporary general-purpose large language models evaluated in the recent medical literature [17,18]. Question retrieval was performed on 21 December 2025. Responses were generated between 22 and 24 December 2025, using ChatGPT-5.2 (OpenAI), Claude Sonnet 4.5 (Anthropic), and DeepSeek-V3.2 through their official, publicly accessible web-based interfaces. All models were accessed through their publicly available free web interfaces using the default user configuration available during the study period. No additional reasoning mode or prompt optimization was applied, and web search or external retrieval functions were not enabled. Responses were generated in independent sessions using identical prompts. Subsequent evaluations, including reliability, quality, readability, accuracy, and hallucination assessments, were conducted between 25 December 2025 and 22 February 2026.

### 3.2. Evaluation Instruments

#### 3.2.1. Reliability Assessment (mDISCERN)

The reliability of AI-generated responses was assessed using the modified DISCERN (mDISCERN) instrument, adapted from the original DISCERN tool developed to evaluate the quality and trustworthiness of consumer health information [19]. The instrument assesses multiple domains, including clarity of aims, achievement of objectives, relevance, transparency of information sources, reporting of publication dates, balance and impartiality, provision of additional resources, and acknowledgment of uncertainty. Each item is scored on a 5-point scale, yielding a total score ranging from 8 to 40, with higher scores indicating greater reliability.

#### 3.2.2. Quality Assessment (GQS)

Overall response quality was evaluated using the Global Quality Scale (GQS), a 5-point instrument commonly used to assess the quality, flow, and usefulness of online health information from a patient perspective [20]. Higher scores indicate greater comprehensiveness and educational value.

#### 3.2.3. Readability Assessment (FRES)

Readability was assessed using the Flesch Reading Ease Score (FRES), a widely used measure of textual readability based on sentence length and word complexity. FRES values were calculated using the Readability Formulas online calculator [21]. According to established recommendations for patient education materials, texts corresponding to approximately the sixth-grade reading level are considered appropriate for the general population. Therefore, higher FRES values indicate greater readability and better accessibility for a broader audience.

#### 3.2.4. Accuracy Assessment

The factual accuracy of each response was evaluated using a 5-point Likert-type scale adapted from our previously published methodology for assessing AI-generated responses to dermatological questions [5]. Scores ranged from 1 (completely inaccurate or misleading information) to 5 (fully accurate information consistent with current evidence-based recommendations). Intermediate scores reflected varying degrees of accuracy and completeness.

#### 3.2.5. Evaluator Roles

All assessments were conducted by researchers according to their areas of expertise. Reliability (mDISCERN), quality (GQS), and accuracy evaluations were performed by a board-certified dermatologist (E.B.). Readability analyses (FRES) were conducted by a public health specialist (M.T.U.). Reference verification and hallucination analyses were independently performed by a public health nursing specialist (E.D.). To ensure transparency and reproducibility, all prompts and AI-generated responses are provided as Supplementary Table S2. Each evaluator assessed only the outcome assigned to his/her area of expertise using predefined scoring criteria. For the dermatologist’s evaluations, all AI-generated responses were anonymized, and model identifiers were removed before assessment; therefore, the dermatologist was blinded to the identity of the AI model that generated each response. Because each evaluation domain was performed by a single designated expert, inter-rater reliability analyses were not applicable.

### 3.3. Hallucination Rate

To assess the reliability of the provided references, a manual verification process was conducted using PubMed (National Library of Medicine, Bethesda, MD, USA) and Google Scholar (Google LLC, Mountain View, CA, USA). References were classified as verifiable, inaccurate, or hallucinated. A reference was considered inaccurate when the original publication could be identified but one or more bibliographic details (e.g., author name, title, journal, or publication year) were reported incorrectly. A reference was classified as hallucinated when no corresponding publication could be identified despite verification attempts. The hallucination rate was calculated as the percentage of hallucinated references relative to the total number of references provided by each model [22]. Overall, 298 AI-generated references were manually evaluated, including 43 references from Claude Sonnet 4.5, 152 from ChatGPT-5.2, and 103 from DeepSeek-V3.2.

All generated references and corresponding outputs are included in the Supplementary Materials to ensure transparency and reproducibility. To improve reporting transparency, the methodological characteristics of the present study are summarized according to the proposed METRICS framework (Table 2) [23].

## 4. Results

### 4.1. Reference Transparency and Hallucination Analysis

Claude Sonnet 4.5 refrained from providing specific references in 14 of the 20 questions. Of the six questions with cited references, one exhibited hallucination (12.5%), while the remaining five contained accurate references. ChatGPT-5.2 provided explicit references for all 20 questions. Hallucination was detected in several responses, with hallucination rates ranging from 18.2% to 50.0% across the evaluated questions. DeepSeek-V3.2 provided references for all questions, with hallucination rates ranging from 10.0% to 58.3% across the evaluated responses. While Claude Sonnet 4.5 refrained from citing sources in most questions, ChatGPT-5.2 and DeepSeek-V3.2 consistently provided references but exhibited higher and more variable hallucination rates across the evaluated items.

### 4.2. Modified DISCERN (mDISCERN)

The mean modified DISCERN (mDISCERN) score was 25.60 ± 5.01 for Claude Sonnet 4.5, 31.45 ± 2.93 for ChatGPT-5.2, and 31.95 ± 3.52 for DeepSeek-V3.2.

A Friedman test was conducted to compare mDISCERN scores among the three AI models and revealed a statistically significant difference (χ^2^(2) = 13.169, *p* = 0.001). The corresponding Kendall’s coefficient of concordance indicated a moderate effect size (W = 0.329). The mean ranks were 1.35 for Claude Sonnet 4.5, 2.33 for ChatGPT-5.2, and 2.33 for DeepSeek-V3.2.

Following the Friedman test, pairwise comparisons of modified DISCERN (mDISCERN) scores among the three AI models were performed using the Wilcoxon signed-rank test with Bonferroni correction (adjusted α = 0.0167). The comparison between ChatGPT-5.2 and Claude Sonnet 4.5 demonstrated a statistically significant difference (Z = −3.306, *p* < 0.001). Similarly, a statistically significant difference was observed between DeepSeek-V3.2 and Claude Sonnet 4.5 (Z = −3.384, *p* < 0.001). In contrast, no statistically significant difference was detected between DeepSeek-V3.2 and ChatGPT-5.2 (Z = −0.523, *p* = 0.601).

### 4.3. Global Quality Score (GQS)

The mean Global Quality Score (GQS) was 3.55 ± 0.60 for Claude Sonnet 4.5, 4.20 ± 0.62 for ChatGPT-5.2, and 4.55 ± 0.60 for DeepSeek-V3.2.

A Friedman test was conducted to compare GQS values among the three AI models and revealed a statistically significant difference (χ^2^(2) = 20.327, *p* < 0.001). The corresponding Kendall’s coefficient of concordance indicated a large effect size (W = 0.508). The mean ranks were 1.38 for Claude Sonnet 4.5, 2.08 for ChatGPT-5.2, and 2.55 for DeepSeek-V3.2.

Following the Friedman test, pairwise comparisons were performed using the Wilcoxon signed-rank test with Bonferroni correction (adjusted α = 0.0167). The comparison between ChatGPT-5.2 and Claude Sonnet 4.5 showed a statistically significant difference (Z = −2.804, *p* = 0.005). A statistically significant difference was also observed between DeepSeek-V3.2 and Claude Sonnet 4.5 (Z = −3.466, *p* < 0.001). In contrast, no statistically significant difference was detected between DeepSeek-V3.2 and ChatGPT-5.2 (Z = −2.333, *p* = 0.020).

### 4.4. Flesch Reading Ease Score (FRES)

The mean Flesch Reading Ease Score (FRES) was 32.05 ± 10.80 for Claude Sonnet 4.5, 47.60 ± 10.84 for ChatGPT-5.2, and 31.65 ± 10.13 for DeepSeek-V3.2.

A Friedman test demonstrated a statistically significant difference in FRES values among the three AI models (χ^2^(2) = 27.300, *p* < 0.001). The corresponding Kendall’s coefficient of concordance indicated a large effect size (W = 0.683). The mean ranks were 1.45 for Claude Sonnet 4.5, 2.95 for ChatGPT-5.2, and 1.60 for DeepSeek-V3.2.

Following the Friedman test, pairwise comparisons were performed using the Wilcoxon signed-rank test with Bonferroni correction (adjusted α = 0.0167). A statistically significant difference was observed between ChatGPT-5.2 and Claude Sonnet 4.5 (Z = −3.921, *p* < 0.001). No statistically significant difference was detected between DeepSeek-V3.2 and Claude Sonnet 4.5 (Z = −0.075, *p* = 0.940). In contrast, a statistically significant difference was found between DeepSeek-V3.2 and ChatGPT-5.2 (Z = −3.830, *p* < 0.001).

### 4.5. Accuracy 

The mean accuracy assessment score was 3.90 ± 0.45 for Claude Sonnet 4.5, 4.35 ± 0.49 for ChatGPT-5.2, and 4.40 ± 0.50 for DeepSeek-V3.2.

A Friedman test revealed a statistically significant difference in accuracy assessment scores among the three AI models (χ^2^(2) = 10.878, *p* = 0.004). The corresponding Kendall’s coefficient of concordance indicated a small-to-moderate effect size (W = 0.272). The mean ranks were 1.58 for Claude Sonnet 4.5, 2.15 for ChatGPT-5.2, and 2.28 for DeepSeek-V3.2.

Following the Friedman test, pairwise comparisons were conducted using the Wilcoxon signed-rank test with Bonferroni correction (adjusted α = 0.0167). A statistically significant difference was observed between ChatGPT-5.2 and Claude Sonnet 4.5 (Z = −2.460, *p* = 0.014). Similarly, a statistically significant difference was found between DeepSeek-V3.2 and Claude Sonnet 4.5 (Z = −3.162, *p* = 0.002). In contrast, no statistically significant difference was detected between DeepSeek-V3.2 and ChatGPT-5.2 (Z = −0.302, *p* = 0.763).

**Figure 2:**
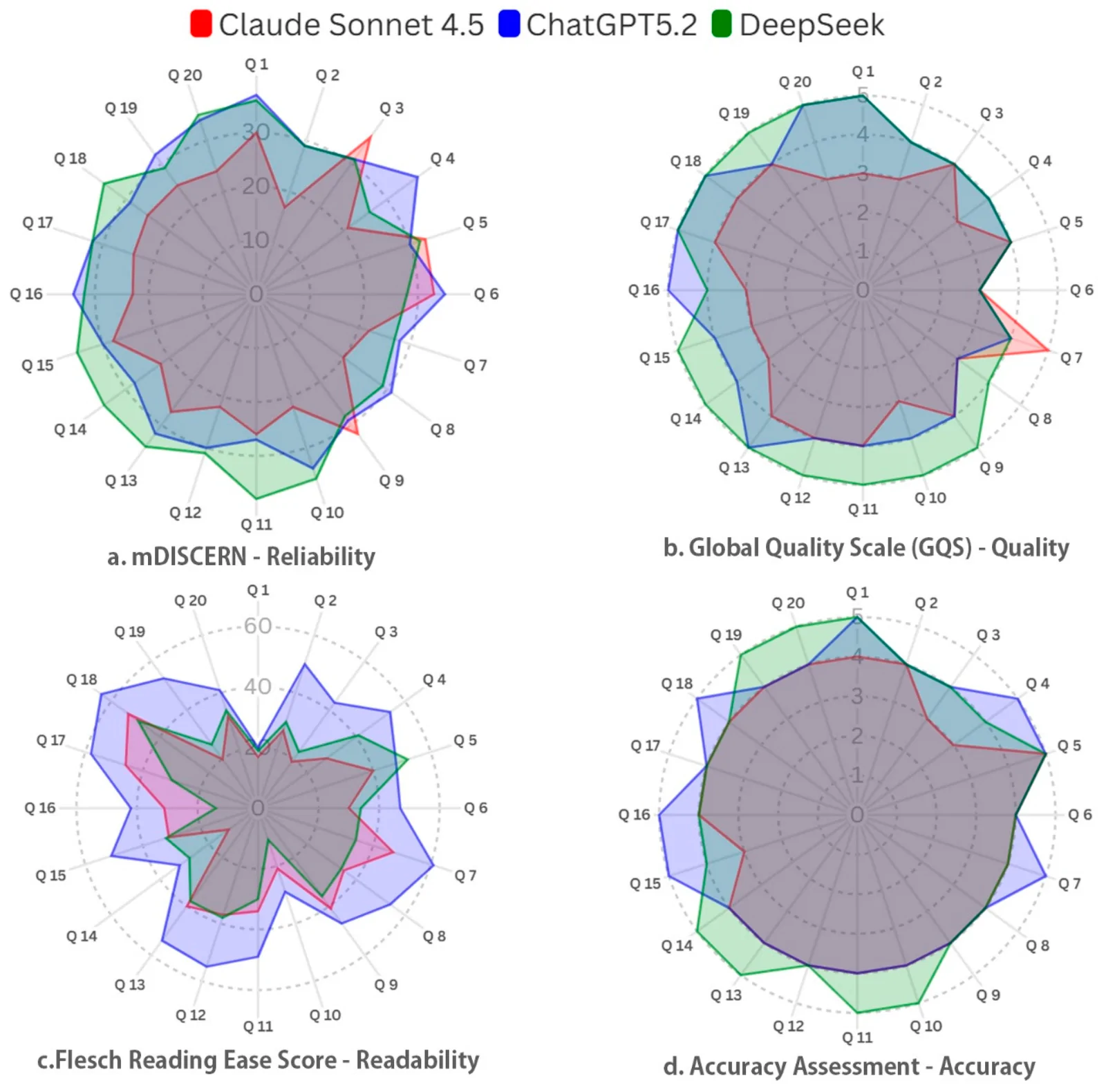
Radar plots of chatbot responses for reliability (mDISCERN), quality (GQS), readability (FRES), and accuracy across the 20 rosacea-related questions. Higher scores indicate better performance in the corresponding evaluation domain.

**Figure 3:**
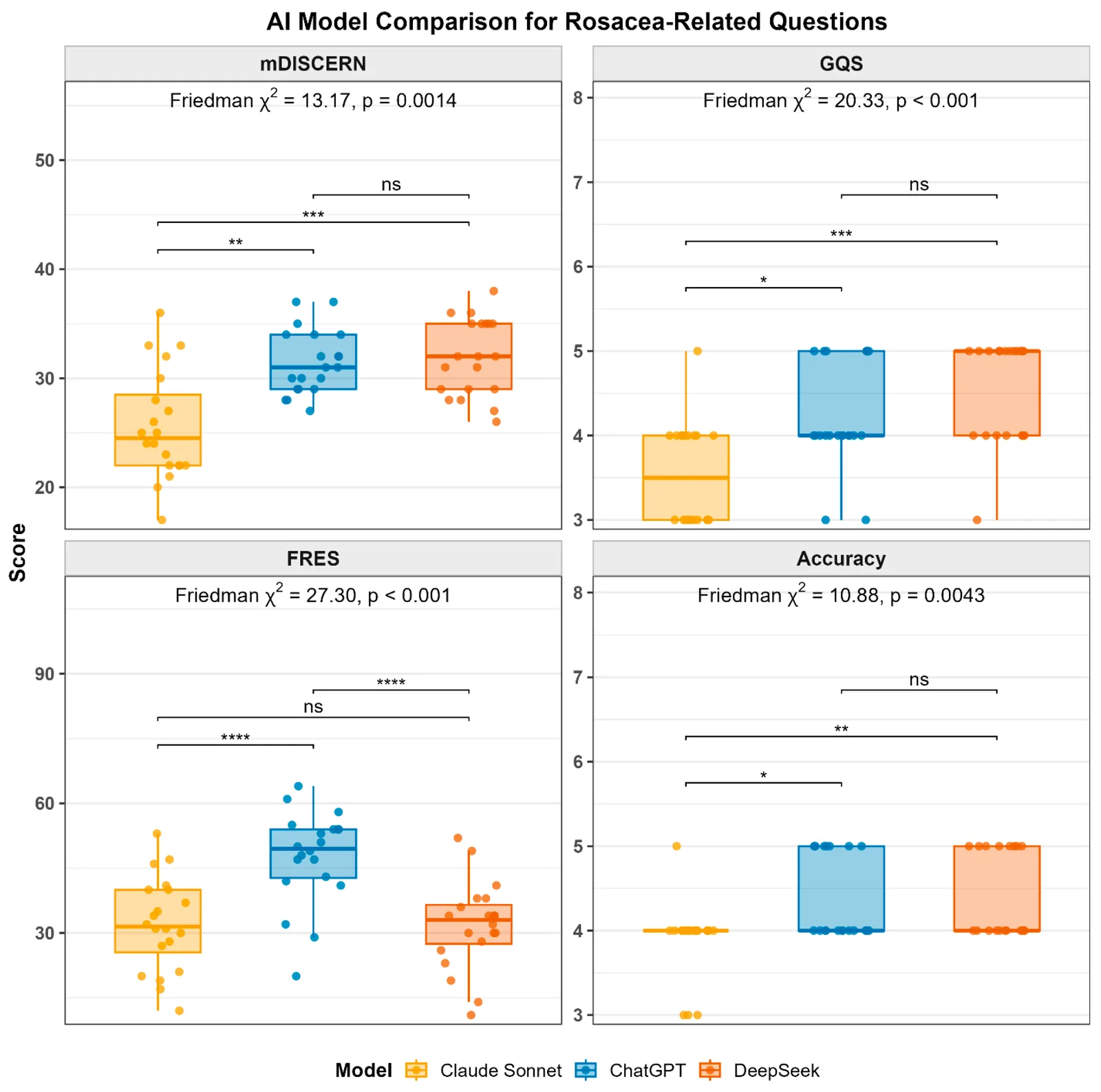
Comparative distribution of mDISCERN, GQS, FRES, and accuracy scores for Claude Sonnet 4.5, ChatGPT-5.2, and DeepSeek-V3.2. Higher scores indicate greater reliability, quality, readability, and accuracy, respectively. Differences were evaluated using the Friedman test with Bonferroni-adjusted post hoc pairwise comparisons. Asterisks indicate statistical significance in pairwise comparisons (* *p* ≤ 0.05, ** *p* ≤ 0.01, *** *p* ≤ 0.001, **** *p* ≤ 0.0001); ns, not significant.

The radar plots of chatbot responses for reliability, quality, readability, and accuracy for the 20 rosacea-related questions are shown in Figure 2, whereas pairwise Wilcoxon signed-rank test results for AI models across evaluation domains are depicted in Table 3. Friedman plots of the chatbots’ answers for mDISCERN, GQS, FRES, and accuracy scores are shown in Figure 3.

## 5. Discussion

The present study compared the performance of three contemporary generative AI chatbots in providing rosacea-related diagnostic and therapeutic information in response to common patient questions. Significant differences were observed among the evaluated models. ChatGPT-5.2 and DeepSeek-V3.2 achieved significantly higher reliability and accuracy scores than Claude Sonnet 4.5. DeepSeek-V3.2 demonstrated the highest overall information quality, whereas ChatGPT-5.2 generated the most readable responses according to the Flesch Reading Ease Score (FRES). These findings highlight the growing potential of generative AI systems as sources of health-related information while underscoring important differences in their performance. However, the present study evaluated only the informational characteristics of AI-generated responses, including reliability, quality, readability, accuracy, and reference transparency. Clinical effectiveness, patient outcomes, and real-world decision-making impacts were not assessed and therefore remain uncertain. Although mDISCERN, GQS, and accuracy assessments are related measures, they evaluate different aspects of AI-generated health information. mDISCERN primarily assesses reliability and transparency, while GQS focuses on overall quality and educational usefulness, and the accuracy assessment evaluates factual correctness. Therefore, the combined use of these instruments provides a more comprehensive evaluation of chatbot performance, although some degree of conceptual overlap cannot be completely excluded.

The higher readability scores observed for ChatGPT-5.2 may facilitate patient comprehension by presenting information in a more accessible manner. In contrast, DeepSeek-V3.2 achieved the highest GQS scores, suggesting that its responses were generally more comprehensive and informative. Although DeepSeek-V3.2 and ChatGPT-5.2 demonstrated comparable reliability according to mDISCERN scores, both substantially outperformed Claude Sonnet 4.5. These findings suggest that different AI-based models may possess distinct strengths, with some prioritizing readability and patient accessibility, while others provide more detailed and comprehensive information. According to current health literacy recommendations, patient education materials should ideally be written at approximately the sixth-grade reading level to ensure accessibility for the general population. Therefore, readability should be considered alongside information quality and accuracy when evaluating AI-generated health information. Similar concerns have recently been reported for AI-generated information on other common musculoskeletal disorders. Despite improvements in response quality, AI-generated patient education materials frequently remain above the recommended readability threshold, highlighting the continuing need for plain-language optimization to ensure accessibility for individuals with different educational and socioeconomic backgrounds [24].

Our findings should also be interpreted in the context of studies conducted in non-English languages. For example, an evaluation of Turkish internet-based patient education materials for low back pain reported moderate readability but low reliability and poor information quality, indicating that challenges related to online health information are not limited to English-language resources [25]. Together, these findings highlight the importance of evaluating AI-generated health information across different languages and healthcare settings.

AI-based chatbots should be considered as part of the broader digital health information ecosystem rather than as isolated tools. Social media platforms, websites, and video-sharing services such as YouTube remain among the most widely used sources of dermatology-related information and patient education. These platforms provide opportunities to improve health literacy and public engagement; however, they also facilitate the rapid dissemination of inaccurate or misleading information. Consequently, ensuring the quality, reliability, and readability of health information across all digital platforms remains essential, and AI-generated content should complement rather than replace evidence-based patient education provided by healthcare professionals [26,27].

Although statistically significant differences were observed among the evaluated models, the practical significance of these findings should be interpreted within the context of their intended use. Even relatively small differences in reliability, quality, or readability may influence patient understanding and the overall usefulness of AI-generated health information. While mDISCERN, GQS, and accuracy assessments evaluate related aspects of information quality, each instrument captures a distinct construct. The mDISCERN instrument primarily assesses the reliability and trustworthiness of information, including transparency, balance, and source-related characteristics. In contrast, GQS evaluates the overall educational value, flow, and usefulness of the content from a user-oriented perspective. Accuracy assessment specifically focuses on the factual correctness and consistency of the information with current evidence-based medical knowledge. Therefore, these instruments should be considered complementary rather than interchangeable. The use of multiple evaluation tools enabled a more comprehensive assessment of AI-generated responses and reduced the risk of relying on a single dimension of performance. Nevertheless, some conceptual overlap between the instruments is acknowledged and represents an inherent limitation of multidimensional content evaluation frameworks.

As AI platforms are becoming increasingly embedded into our daily lives, patients prefer to direct their common queries related to different health problems to generative AI tools. Therefore, it is crucial to comprehend the impact of AI tools on patient decision-making and behavior [28]. As patients often consult physicians after obtaining health information from online sources and AI-based platforms, physicians need to clarify and validate the knowledge derived from digital content [28]. In this context, it seems to be quite significant to evaluate the accuracy, readability, reliability, and quality of different AI platforms in providing different health-related information. As digital health information-seeking behavior continues to increase, further studies are needed to validate information generated by AI tools.

Skin-related problems are likely to comprise a considerable proportion of consultations to AI platforms. Rosacea is a chronic inflammatory skin disease that predominantly affects the face in fair-skinned individuals and may have a considerable impact on the patient’s quality of life. Rosacea is reported to have a global prevalence of 1–3% [6]. Therefore, many patients may seek information about the pathogenesis, clinical features, and treatment of rosacea through AI-based platforms. Different studies have been conducted to assess the performance of different AI tools in answering questions related to common skin disorders [13,29,30,31]. In a study that evaluated the clinical applicability and reliability of ChatGPT’s responses to common questions about rosacea, it was shown that ChatGPT demonstrated a high reliability rate, which ranged from 92.22% to 97.78%, whereas the clinical applicability rate ranged from 98.61% to 100.00% [13]. Overall, these findings suggest that ChatGPT 3.5 is likely to be an excellent tool for patient education about rosacea. Another recent study by Nelson et al. [27], which compared the readability, quality, and reliability of rosacea-related health information between traditional search tools (Google and Bing) and Gemini and ChatGPT, revealed that Google demonstrated the highest mean scores for both quality and reliability. By contrast, ChatGPT had the lowest scores for all quality parameters [27]. The results of the study underlined the fact that Google was the most credible source of qualified and legible data among all handled platforms [27]. Another study by Chau et al. [32] evaluated the competence of ChatGPT-3.5, Bing AI, and Google Bard in answering questions about 5 common dermatological diseases (rosacea, atopic dermatitis, acne, cysts, and actinic keratosis). ChatGPT’s answers showed significantly higher accuracy in comparison to other chatbots, while lower comprehensibility was detected by FRES in ChatGPT’s responses [32]. In our study, DeepSeek-V3.2 received the highest score, followed by ChatGPT-5.2 and Claude Sonnet 4.5 for quality, whereas ChatGPT-5.2 had the highest readability scores, followed by Claude Sonnet 4.5 and DeepSeek-V3.2.

AI-based diagnostic models have also been developed to predict Demodex mite density in patients with facial erythema [33]. In this study, the created deep learning model, DemodexNet, achieved encouraging performance in detecting Demodex mite density and enhancing the diagnostic accuracy of dermatologists [33]. Diagnostic performance of ChatGPT-4.0 and Gemini Flash 2.0 in recognizing rosacea and acne images was also evaluated; ChatGPT-4.0 had an accurate diagnostic rate of 93%, while Gemini Flash 2.0 provided a diagnosis for 21% of the cases [34]. However, ChatGPT-4.0 achieved moderate success in rosacea subtyping with 50% sensitivity and 80% specificity [34]. Another novel convolutional neural network was tested to differentiate between clinical images of rosacea from other cutaneous diseases such as acne and seborrheic dermatitis [35]. The outcomes of this study underlined that the developed convolutional neural network reached adequate precision and accuracy, which was similar to that of an experienced dermatologist [35].

Natural language processing (NLP) is a field within AI and computer science that uses machine learning algorithms to enable computers to process and communicate using human language [36]. A recent systematic review by Paganelli et al. [36] investigated the applications of NLP in dermatology through a literature search of MEDLINE and Scopus databases. The results showed that NLP tools have been used for different dermatologic problems such as atopic dermatitis, rosacea, acne, skin cancers, and skin infections [36]. It is concluded that although NLP offers valuable opportunities to improve dermatology research and clinical care, addressing challenges related to data accuracy, consistency, interpretation, and patient privacy remains essential.

It is also significant to evaluate the performance of different AI tools in terms of patient safety implications, as inappropriate medical recommendations or treatments may lead to delayed referrals and potentially detrimental outputs. A recent study by Draelos et al. [7], which evaluated the responses of four different AI chatbots (Meta Llama, Anthropic Claude, Google Gemini, and OpenAI GPT-4o) to medical-advice-seeking queries of the patients, showed that problematic response rates ranged from 21.6% for Claude to 43.2% for Llama, while unsafe responses ranged from 5% for Claude to 13% for GPT-4o and Llama. Another narrative review by Omiye et al. [37] highlighted the fact that even though large language models show promising results in the medical field, they are susceptible to exhibiting hallucinations and diagnostic inaccuracies that may compromise patient safety. In the present study, the hallucination rate was utilized to evaluate the credibility of the provided references by ChatGPT-5.2, DeepSeek-V3.2, and Claude Sonnet-4.5. Hallucination rate refers to the ratio of fabricated references to all references. Claude Sonnet-4.5 did not provide specific references for 70% of the evaluated questions, whereas DeepSeek-V3.2 and ChatGPT-5.2 consistently provided references, but the hallucination rates ranged from 10.0% to 58.3% for DeepSeek-V3.2 and 18.2% to 50.0% for ChatGPT-5.2. Chelli et al. [22] assessed the hallucination rates of ChatGPT-3.5, ChatGPT-4, and Bard using researcher-conducted systematic reviews. The hallucination rates were 39.6% for ChatGPT-3.5, 28.6% for ChatGPT-4, and 91.4% for Bard (*p* < 0.001). These findings are consistent with recent evidence indicating that hallucinations in large language models should not be viewed merely as factual inaccuracies but also as a potential patient safety concern, as fabricated or misleading outputs may adversely influence health-related decision-making. Accordingly, future mitigation strategies should focus not only on improving factual accuracy but also on reducing the potential safety risks associated with AI-generated medical information [38]. In concordance with the results of our study, it is important to once again underline the requirement for rectifying the reliability and functionality of large language models before they can be used reliably for academic purposes. With the increasing integration of AI into healthcare, it is highly important for practitioners to be familiar with their design, capabilities, emerging uses, and associated risks.

In the present study, a statistically significant difference was found across different AI chatbots in terms of FRES value, which is mainly an indicator of readability; ChatGPT-5.2 had the highest FRES value, followed by Claude Sonnet 4.5 and DeepSeek-V3.2. A systematic meta-synthesis of 42 studies assessed chatbot responses by using the DISCERN instrument and measured readability levels [39]. The results pointed to the fact that as the quality of chatbot responses increased, their readability generally decreased; of the 86 tests, only 49 produced responses that were rated as “good” quality or better, but only 10 of them achieved readability levels below college level [39]. The current quality and readability of chatbot-generated medical information seem to be insufficient for patient use. However, prompt engineering may improve both readability and quality, thereby better meeting patients’ needs and enabling more effective communication with diverse patient populations.

Despite the promising performance of contemporary AI chatbots, these systems should be regarded as complementary tools rather than substitutes for professional medical care. AI-generated responses cannot replace a comprehensive face-to-face clinical evaluation, physical examination, individualized risk assessment, or clinical judgment provided by qualified healthcare professionals. A balanced approach that combines the technical capabilities of AI with patient-centered clinical care remains essential to ensure safe and appropriate healthcare decision-making [22,23].

## 6. Limitations

Several limitations should be considered when interpreting the findings of this study. First, the analysis was limited to rosacea-related questions; therefore, the findings may not be generalizable to other dermatological conditions or medical specialties.

Second, only three contemporary large language models (ChatGPT-5.2, Claude Sonnet 4.5, and DeepSeek-V3.2) were evaluated. Although these models are among the most widely used, the findings may not fully represent the performance of other available AI systems. In addition, responses were generated and evaluated at a single time point. Because large language models are continuously updated and exhibit stochastic behavior, their performance and responses may vary across different sessions, model updates, or time points. Consequently, the present findings reflect the performance of the evaluated models during the study period.

Third, all expert evaluations were performed by a single board-certified dermatologist; therefore, inter-rater variability could not be assessed. Although validated assessment instruments were used to evaluate reliability, quality, readability, and accuracy, some degree of evaluator subjectivity may have influenced the scoring process. In addition, readability was assessed using a single online calculator. Although this approach is commonly used, minor variations may occur between different readability calculators due to differences in implementation; therefore, the reported readability scores should be interpreted accordingly. Future studies involving multiple independent dermatologists may further strengthen the robustness of subjective evaluations.

Fourth, all responses were generated and evaluated in English. Therefore, linguistic and cultural differences may limit the applicability of the findings to other languages and healthcare settings.

Finally, this study focused exclusively on text-based responses and did not evaluate multimodal capabilities, such as image interpretation, which are increasingly incorporated into contemporary AI systems. Furthermore, the hallucination analysis was restricted to reference hallucinations. Other clinically important hallucination types—including factual inaccuracies, contradictory treatment recommendations, flawed clinical reasoning, and truth hallucinations—were not systematically evaluated. Future studies should adopt multidimensional hallucination assessment frameworks to provide a more comprehensive evaluation of AI-generated medical information.

### Strengths

This study has several strengths. To our knowledge, it is the first study to comprehensively compare ChatGPT-5.2, Claude Sonnet 4.5, and DeepSeek-V3.2 in providing responses to rosacea-related patient questions. Multiple complementary outcome measures, including reliability (mDISCERN), quality (GQS), readability (FRES), accuracy, and reference hallucinations, were evaluated using a standardized methodology. In addition, identical prompts, independent chat sessions, and a two-step assessment strategy were used to improve methodological consistency and transparency.

## 7. Conclusions

Generative AI chatbots are increasingly being used as accessible sources of healthcare information, including information related to symptom recognition, disease management, and treatment options. In this study, significant differences were observed among ChatGPT-5.2, Claude Sonnet 4.5, and DeepSeek-V3.2 in terms of reliability, quality, readability, accuracy, and reference transparency. While ChatGPT-5.2 and DeepSeek-V3.2 demonstrated strong overall performance, substantial variability in citation practices and reference hallucination rates was identified. These findings suggest that generative AI systems may have potential as complementary sources of healthcare information; however, their role as decision-support tools requires further validation before routine clinical implementation.

Although online platforms and AI-based chatbots have considerable potential to improve access to health information and support patient education, they should not be used independently as the primary source of medical information or for clinical decision-making. In critical aspects of patient care, including individualized treatment recommendations, risk–benefit assessment, and recognition of red-flag symptoms, AI-generated information remains inherently limited when compared with evidence-based clinical guidelines and physician judgment. Therefore, AI should be regarded as a complementary educational resource that supports, rather than replaces, professional medical consultation.

## Figures and Tables

**Table 1:** Rosacea-related questions retrieved from Quora and asked to Claude Sonnet 4.5, ChatGPT-5.2, and DeepSeek-V3.2.

Questions
Are there any new treatments for rosacea?
Can rosacea occur only on one cheek? What is the cause of it?
Can vitamin A help with rosacea, and if so, why?
Does going gluten-free help with rosacea?
What is the best laser treatment for rosacea?
What causes rosacea?
What is the difference between a lupus rash and rosacea?
What is the difference between acne and rosacea?
How do you reduce rosacea flare-ups?
Is rosacea an autoimmune disease?
Is rosacea caused by diet?
How do I have less redness from rosacea?
How long does rosacea last?
What are the most commonly prescribed creams for treating rosacea?
Has anyone healed their rosacea?
What are the dangers of rosacea?
What are the symptoms of rosacea?
What foods trigger rosacea?
What is the cure for erythematotelangiectatic rosacea?
When can the redness caused by rosacea be considered permanent?

**Table 2:** Alignment of the present study with the proposed METRICS framework for generative AI chatbot evaluation.

METRICS Component	Present Study
Model	ChatGPT-5.2, Claude Sonnet 4.5, DeepSeek-V3.2
Evaluation	Accuracy, mDISCERN, GQS, FRES, hallucination analysis
Timing	Question retrieval: 21 December 2025; Response generation: 22–24 December 2025
Transparency	Questions derived from Quora; selection procedure described
Range	20 representative rosacea questions covering diagnosis, triggers, treatment, skincare, and disease manifestations
Randomization	Not performed; representative sampling was intentionally used
Individual factors	Domain-specific expert evaluation; reliability (mDISCERN), quality (GQS), and accuracy assessed by one board-certified dermatologist; readability and reference verification performed by designated public health experts.
Count	20 questions × 3 chatbots = 60 responses evaluated
Specificity	Identical prompts; English language; prompts included in Supplementary Materials

**Table 3:** Pairwise Wilcoxon signed-rank test results for AI models across evaluation domains.

Evaluation Domain	Compared Models	Z Value	*p* Value (2-Tailed)
mDISCERN	Claude Sonnet 4.5 vs. ChatGPT-5.2	−3.31	**<0.001**
mDISCERN	Claude Sonnet 4.5 vs. DeepSeek-V3.2	−3.38	**<0.001**
mDISCERN	ChatGPT-5.2 vs. DeepSeek-V3.2	−0.523	0.601
GQS	Claude Sonnet 4.5 vs. ChatGPT-5.2	−2.80	**0.005**
GQS	Claude Sonnet 4.5 vs. DeepSeek-V3.2	−3.466	**0.001**
GQS	ChatGPT-5.2 vs. DeepSeek-V3.2	−2.33	0.020
FRES	Claude Sonnet 4.5 vs. ChatGPT-5.2	−3.92	**<0.001**
FRES	Claude Sonnet 4.5 vs. DeepSeek-V3.2	−0.07	0.940
FRES	ChatGPT-5.2 vs. DeepSeek-V3.2	−3.83	**<0.001**
Accuracy	Claude Sonnet 4.5 vs. ChatGPT-5.2	−2.46	**0.014**
Accuracy	Claude Sonnet 4.5 vs. DeepSeek-V3.2	−3.16	**0.002**
Accuracy	ChatGPT-5.2 vs. DeepSeek-V3.2	−0.30	0.763

Pairwise comparisons were performed using the Wilcoxon signed-rank test with Bonferroni correction for multiple testing. Significant comparisons after Bonferroni correction are shown in bold. Results were considered statistically significant at *p* < 0.0167. Global effect sizes for the corresponding Friedman analyses were mDISCERN W = 0.329, GQS W = 0.508, FRES W = 0.683, and accuracy W = 0.272.

## Data Availability

The original contributions presented in this study are included in the article/Supplementary Materials. Further inquiries can be directed to the corresponding author.

## Supplementary Materials

The following supporting information can be downloaded at: https://www.mdpi.com/article/10.3390/diagnostics16162620/s1, Supplementary Table S1. Shortlisted Candidate Rosacea-Related Questions and Their Selection for Final Analysis; Supplementary Table S2. Complete AI-Generated Responses, Evaluation Scores, and Reference Verification Results for the 20 Rosacea-Related Questions.
